# The acetylation mechanism and engineering strategy of isocitrate lyase for optimizing biosynthesis in *Escherichia coli*

**DOI:** 10.1016/j.synbio.2026.04.031

**Published:** 2026-05-23

**Authors:** Ruoyu Jia, Jichao Wang, Ying Zhang, Mo Xian, Qingsheng Qi, Min Liu, Guang Zhao

**Affiliations:** a State Key Laboratory of Microbial Technology, Shandong University, 266237, Qingdao, China; b CAS Key Laboratory of Biobased Materials, Qingdao Institute of Bioenergy and Bioprocess Technology, Chinese Academy of Sciences, Qingdao, 266101, China

**Keywords:** Lysine acetylation, Isocitrate lyase, Enzyme activity, Protein stability, Glyoxylate shunt

## Abstract

Lysine acetylation is a widespread post-translational modification that fine-tunes cell physiology and metabolism. Yet its application in metabolic engineering remains underexplored. Here, we report that the key enzyme of the glyoxylate shunt, isocitrate lyase (AceA) in *Escherichia coli*, undergoes reversible lysine acetylation mediated by both enzymatic and non-enzymatic mechanisms. We demonstrated that acetylation significantly inhibits AceA activity. Through structural analysis and site-directed mutagenesis, we identified key lysine acetylation sites that critically influence enzyme activity. Furthermore, we developed an acetylation-mediated rational design strategy, constructing the AceA(S335Q/S398Q) double mutant, which exhibited a 2.12-fold increase in enzymatic activity and enhanced protein stability compared to the wild-type. Application of this optimized AceA variant in engineered *E. coli* strains significantly improved the production of some valuable chemicals by reinforcing the glyoxylate shunt and enhancing carbon conversion efficiency. Our work elucidates the regulatory mechanism of AceA acetylation and establishes protein acetylation as a promising and versatile tool for optimizing metabolic pathways for bioproduction.

## 1 Introduction

Cellular physiology is governed by complex regulatory mechanisms that enable cells to adapt to environmental fluctuations. Post-translational modifications (PTMs) represent a crucial regulatory mechanism that covalently alter amino acids through biochemical reactions, thereby enhancing protein structural complexity and refining regulatory precision [1,2]. Over the past 50 years, more than 200 types of protein modifications have been discovered, such as phosphorylation, ubiquitination, methylation, and acetylation, etc [3,4]. Among these, the acetylation of lysine in proteins is a highly conserved PTM with broad implications in gene expression, metabolism, and cellular homeostasis [1]. The acetylation of lysine can be catalyzed by lysine acetyltransferases in an enzymatic mechanism or by acetyl phosphate (AcP) in a nonenzymatic mechanism [5]. Both these two kinds of acetylations can be reversed by histone deacetylases (HDACs) or sirtuins (SIRTs) [6].

Lysine acetylation was initially characterized in the context of histone modification and epigenetic regulation [7]. However, the development of proteomics has revealed its widespread presence on non-histone proteins, indicating that acetylation plays a significant role in various physiological and metabolic processes [8,9]. Therefore, lysine acetylation may become a potential tool in metabolic engineering. By manipulating acetylation levels or creating an enzyme that is unaffected by acetylation, we can open new possibilities for improving bioproduction. However, the regulation of post-translational modification levels has not received much attention in this field.

**Fig. 1:**
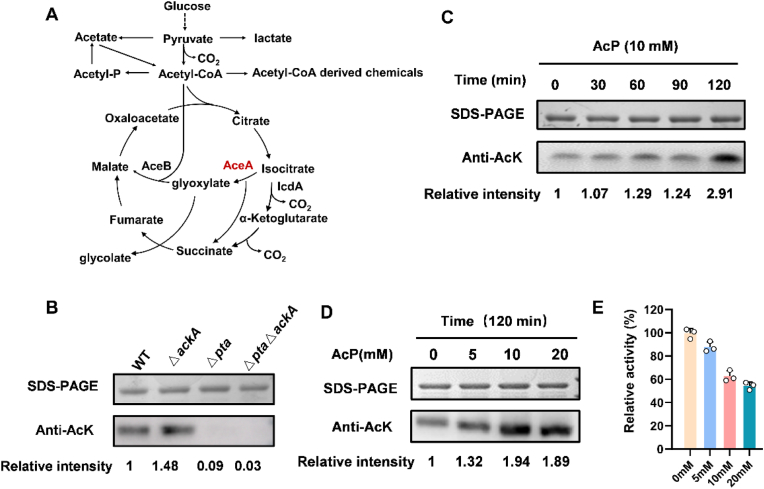
Non-enzymatic acetylation of AceA in *E. coli*. (A) The metabolic pathway catalyzed by AceA and other enzymes. (B) The acetylation levels of AceA in Δ*pta*, Δ*ackA,* and their corresponding double mutant strain. (C) Dose-dependent acetylation of AceA after treatment with AcP in *vitro.* (D) Time-dependent acetylation of AceA after treatment with AcP in *vitro*. (E) Effect of AcP treatment for varying concentrations on AceA activity.

The isocitrate lyase (AceA) catalyzes the first step of the glyoxylate shunt in *Escherichia coli*, enabling the assimilation of simple carbon sources such as acetate for net synthesis of biomass and target products (Fig. 1A) [10]. The glyoxylate shunt can bypass the two decarboxylation reactions of TCA cycle, reducing the depletion of carbon source and replenishing the pool of TCA cycle intermediates [11]. Regulating the glyoxylate shunt through AceA may improve the production of some chemicals and the carbon atom economy. Lysine acetylation provides a precise strategy to regulate the activity, protein levels, and stability of enzymes. The acetylome of *E. coli* has been revealed to show that the AceA protein undergoes acetylation at multiple lysine sites [12,13]. However, the role of lysine acetylation in regulating the protein function of *E. coli* AceA remains unclear.

In this study, we found that *E. coli* AceA can be acetylated by enzymatic and AcP-mediated non-enzymatic mechanisms, and the deacetylase CobB can deacetylate AceA. Furthermore, we investigated the effects of specific acetylation sites on AceA activity and elucidated the underlying regulatory mechanisms. Finally, the rational modulation of AceA activity by lysine acetylation effectively enhanced the production of multiple valuable chemicals. These findings indicated that lysine acetylation-mediated regulation is an efficient strategy in metabolic engineering for improving the synthesis of some chemicals and the carbon conversion efficiency.

## 2 Materials and methods

### 2.1 Construction of plasmids and strains

All plasmids and strains used in this study were listed in Table S1. Standard molecular biology techniques, including PCR and restriction enzyme digestion, were used for plasmid construction, and *E. coli* DH5α was chosen as the host. Protein expression and target production were carried out in *E. coli* BL21(DE3). The site-directed mutagenesis of *aceA* was carried out according to the Stratagene protocol. The chromosomal mutant in *E. coli* BL21(DE3) was constructed through CRISPR and P1 vir-mediated transduction. The donor strains for P1 vir-mediated transduction were obtained from the Keio collection [14].

### 2.2 Expression, purification, and stability analysis of proteins

Strains were grown in LB medium supplemented with appropriate antibiotics. Overnight cultures were diluted 1:50 into fresh LB containing 2% glucose and incubated at 37 °C. At OD600 ≈ 0.6, 100 μM IPTG was added for inducing protein expression, followed by incubation at 30 °C for 18 h. Cells were harvested, resuspended in PBS (pH 7.5), and lysed under high pressure. Supernatants were purified using Ni-NTA His·Bind columns (Novagen) according to the supplier's protocol. Purified proteins were used in acetylation and activity assays.

For protein expression analysis, cells were grown to an OD600 of approximately 0.6, followed by the addition of 100 μM IPTG and incubation at 30 °C. Samples were collected at various time points post-induction. For stability tests, strains were grown in LB with 2% glucose. After 4 h of induction with IPTG, chloramphenicol (200 μg/mL) was added to treat the cultures for different times. Cells were harvested and resuspended in PBS, and disrupted by high-pressure homogenization. After centrifugation, supernatants containing total cellular proteins were collected and normalized for protein concentration. Samples were analyzed via 12% SDS-PAGE and Western blot with anti-His antibody.

### 2.3 Western blotting

Proteins were separated on 12% SDS-PAGE gels and transferred to PVDF membranes at 15 V for 1.5 h. After blocking with a quick block buffer (Beyotime), membranes were probed with an anti-acetyllysine mouse monoclonal antibody (EASYBIO, 1:2000) at 4 °C overnight, followed by incubation with an HRP-conjugated anti-mouse secondary antibody (EASYBIO, 1:10000). Signals were detected using an enhanced chemiluminescence (ECL) system after washing with PBST.

### 2.4 AceA activity assay

The activity of AceA was measured according to a similar protocol as previously described [15,16]. Each reaction mixture contained 50 mM MOPS (pH 6.8), 5 mM MgCl_2_, 1 mM NADH, 7 units of lactate dehydrogenase, 5 mM isocitrate. The activity assays for the acetylaiton site-mimicking mutants were performed using 1 μM purified AceA protein, whereas 0.4 μM was used for all other activity measurements. NADH oxidation was monitored at 340 nm using a Spark microplate reader (Tecan).

### 2.5 In vitro acetylation and deacetylation

Acetyltransferase YfiQ-mediated acetylation and Deacetylase CobB-mediated deacetylation in *vitro* tests were performed based on a previously reported protocol with some modifications [16]. The acetylation reaction mixture consisted of 50 mM Tris–HCl (pH 7.8), 0.1 mM EDTA, 10% glycerol, 1 mM dithiothreitol, 10 mM sodium butyrate, 0.2 mM acetyl-CoA, 100 μg of YfiQ, and 40 μg of AceA in a total volume of 200 μL. The mixture was incubated at 37 °C for 1 h.

The deacetylase reaction system contained 40 mM HEPES (pH 7.0), 6 mM MgCl_2_, 1 mM NAD^+^, 2 mM NAM^+^, 1 mM dithiothreitol, 10% glycerol, 100 μg of CobB, and 40 μg of AceA in a final volume of 200 μL. Incubation was performed at 37 °C for 1 h. All the treated samples were analyzed for Western blot and enzyme assays.

For AcP-mediated acetylation assays, 50 μg of AceA was mixed with different concentrations of AcP in a total volume of 100 μL, followed by incubation at 37 °C for varying times.

### 2.6 Shake-flask fermentation and analytical methods

Shake-flask experiments were performed in triplicate using 250 mL unbaffled Erlenmeyer flasks, each containing 50 mL of fermentation medium. After overnight growth at 37 °C in LB broth, the culture was diluted 1:50 into fresh fermentation medium. When the culture reached an OD600 of about 0.6, 0.1 mM IPTG was added, and incubation was continued at 30 °C.

The growth of cells was quantified by measuring the absorbance of the culture at 600 nm with a Hitachi U-2900 spectrophotometer. The concentrations of residual glucose were determined via high-performance liquid chromatography (HPLC) using an Agilent 1260 Infinity series system equipped with a Bio-Rad HPX-87H column (300 × 7.8 mm). Methods for fermentation and analysis of PG [17], 3HP [18], and glycolate [19] followed previously established protocols. Intracellular AcP concentration was determined at 505 nm via the formation of ferric acetyl-hydroxamate using a hydroxamate-based assay [20].

## 3 Results and discussion

### 3.1 AceA can be acetylated by nonenzymatic and enzymatic mechanism

Acetyl phosphate (AcP) is a metabolic intermediate generated through the phosphotransacetylase (Pta) and acetate kinase (AckA) pathway, and serves as the key acetyl donor, directly acetylating the deprotonated lysine ε-amino group by a non-enzymatic mechanism [21,22]. Intracellular AcP levels increase in the *ackA* mutant, whereas the *pta* mutation results in the reduction of AcP concentration relative to the wild-type strain [23,24]. To evaluate the role of AcP in AceA acetylation in *vivo*, we constructed Δ*pta*, Δ*ackA,* and their corresponding double mutant strain, and analyzed their acetylation profiles using an anti-acetyllysine monoclonal antibody. Consistent with AcP availability, AceA acetylation was markedly decreased in both Δ*pta* and Δ*pta*Δ*ackA* mutants, but increased in the Δ*ackA* background (Fig. 1B). Further in *vitro* assays, incubating purified AceA with increasing concentrations of AcP for varying time resulted in acetylation that was both dose- and time-dependent (Fig. 1C and D).

A lysine acetyltransferase (KAT) in *E. coli* has been identified: YfiQ, which belongs to the GCN5-like acetyltransferase (GNAT) family. In fact, a similar enzyme termed Pat was first discovered and characterized in *Salmonella enterica* [25,26]. The KAT can transfer the acetyl group from the donor acetyl-CoA to the ε-amino group of lysine residues in the target protein [21,22]. In *E. coli*, a NAD-dependent deacetylase CobB serves as the primary lysine deacetylase, exhibiting the ability to remove acetyl groups regardless of their enzymatic or non-enzymatic origin [6,27].

**Fig. 2:**
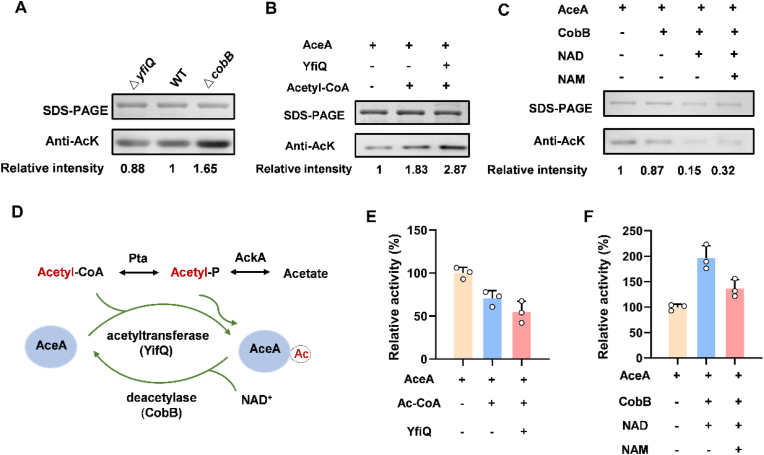
Enzymatic acetylation of AceA protein in *E. coli*. (A) The acetylation levels of AceA in *yfiQ* and *cobB* mutant strains. (B) The acetylation levels of AceA by acetyltransferase YfiQ treatment in *vitro*. (C) The acetylation levels of AceA by deacetylase CobB treatment in *vitro*. (D) Schematic representation of enzymatic and non-enzymatic acetylation mechanisms targeting AceA in *E. coli*. (E) Effects of YfiQ treatment on AceA activity. (F) Effects of CobB treatment on AceA activity.

To investigate the acetylation mechanism of AceA and its potential reversibility in *E. coli*, we also performed Western blot analysis using an anti-acetyllysine monoclonal antibody. Firstly, the roles of YfiQ and CobB for AceA acetylation modification were assessed both in *vivo* and in *vitro*. We constructed *yfiQ* and *cobB* knockout mutants in *E.* *coli*, and the AceA overexpression plasmid was transformed into these mutants, respectively. The acetylation level of AceA decreased in the Δ*yfiQ* strain and increased in the Δ*cobB* strain compared to the wild type (Fig. 2A). We then purified YfiQ and CobB and incubated each with AceA in *vitro*. The acetylation of AceA was enhanced upon incubation with acetyl-CoA and was further enhanced when co-incubated with acetyl-CoA and YfiQ (Fig. 2B). Western blotting suggested that AceA acetylation occurs both enzymatically, catalyzed by YfiQ, and non-enzymatically by acetyl-CoA (Fig. 2B). Additionally, the acetylation level of AceA was significantly reduced upon incubation with the NAD^+^-dependent deacetylase CobB. This deacetylation reaction was inhibited by the addition of nicotinamide (NAM), a known competitive inhibitor of CobB (Fig. 2C).

Collectively, our findings demonstrated that the metabolic enzyme AceA in *E. coli* is subject to both non-enzymatic and enzymatic acetylation. Notably, we provided evidence that this modification is reversible through the catalysis of the sirtuin-like deacetylase CobB (Fig. 2D). The dynamic and reversible modification places AceA within a dual-mode acetylation control. This mechanism underscores the critical physiological importance of AceA regulation at the post-translational level, enabling cells to adapt to a wide range of metabolic states by modulating its activity. It is noteworthy that the reversibility of acetylation in *E. coli* has only been demonstrated for a small number of instances [6]. This observation implies that reversible acetylation is likely a precisely regulated process that plays crucial physiological roles, rather than being a widespread or indiscriminate phenomenon. Another possibility is that specific deacetylases responsible for reversing the acetylation of these CobB-insensitive substrates have not yet been identified. Therefore, research efforts aimed at discovering new deacetylases have continued persistently. For example, the serine hydrolase YcgC was initially proposed as a candidate, though subsequent studies could not confirm its deacetylation activity [28]. In *Mycobacterium smegmatis*, the protein MSMEG_4620 has been reported to exhibit deacetylation activity, suggesting evolutionary conservation of non-sirtuin deacetylases in bacteria [29]. Nevertheless, the field continues to face some challenges in conclusively identifying and characterizing new deacetylases.

### 3.2 Carbon source affects the acetylation status of AceA

**Fig. 3:**
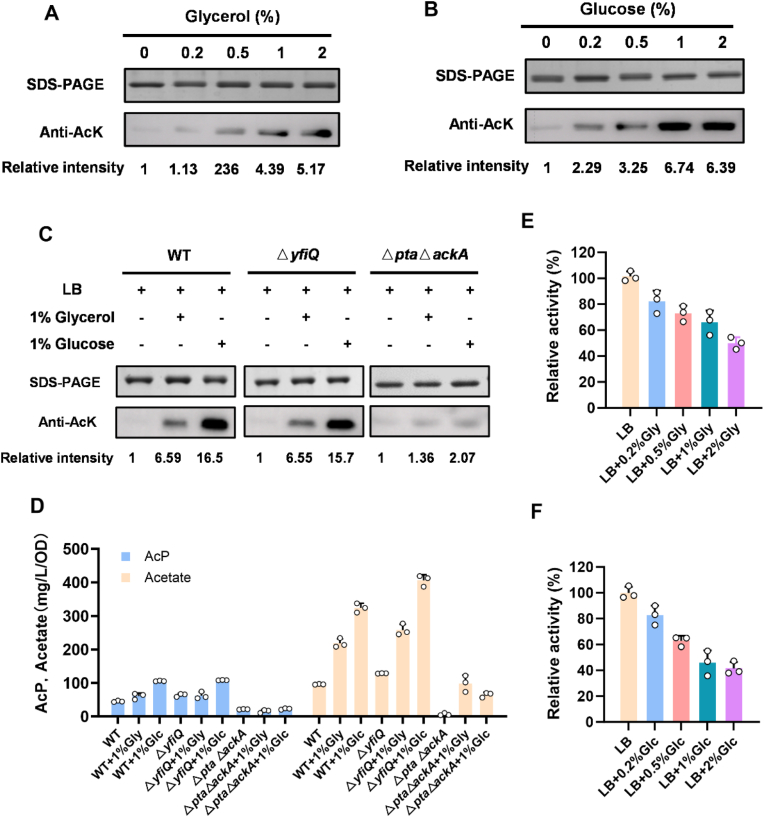
Carbon source affects the acetylation status of AceA. The acetylation status of AceA in LB medium supplemented with varying concentrations of glycerol (A) or glucose (B). (C) The effects of carbon sources on AceA acetylation in wild-type*, yfiQ* knockout, and *pta*-*ackA* double knockout strains. (D) The concentration of AcP and acetate in wild-type, *yfiQ* mutant, and *pta*-*ackA* double mutant strains under varying conditions. (E) Effects of glycerol supplement for varying concentrations on AceA activity. (F) Effects of glucose supplement for varying concentrations on AceA activity. Gly: glycerol; Glc: glucose.

The choice of carbon source in microbial fermentation has a critical influence on both cell growth and product synthesis by modulating metabolic flux and energy generation. The type of carbon source can also affect the cellular acetylome, altering intracellular pools of key metabolic precursors, acetyl-CoA, and acetyl-phosphate [16,30]. Understanding carbon-dependent acetylation dynamics may provide a novel strategy for optimizing fermentation processes, enhancing the synthesis of target products, and reducing undesirable byproduct formation through post-translational regulation without the need for genetic intervention. To investigate the effect of carbon source on AceA acetylation, AceA was purified from cells grown in LB medium supplemented with varying concentrations of glucose or glycerol, and the acetylation levels were assessed by Western blot analysis. The acetylation level of AceA was elevated following supplementation with glucose or glycerol, showing a clear dose-dependent increase, and glucose induces higher acetylation levels than glycerol at equal concentrations (Fig. 3A and B). However, increasing glucose from 1% to 2% did not further elevate the acetylation (Fig. 3B). A concentration of 1% glucose may be sufficient to induce the maximal acetylation of AceA, with no further increase observed at 2% concentration of glucose.

It is important to note that not all carbon sources lead to differences in protein acetylation levels at the same concentrations. Similar acetylation levels were observed in cells grown on the same concentration of xylose versus glucose (Schilling et al., 2019). Schilling et al. proposed that the effects of carbon source on lysine acetylation may arise from variations in the downstream metabolites rather than carbon source identity per se. To investigate the mechanism by which different carbon sources affect AceA acetylation, we constructed mutant strains deficient in enzymatic and non-enzymatic acetylation pathways by deleting the *yfiQ* and *pta*-*ackA* genes, respectively. We assessed the acetylation status of AceA in the wild-type and mutant strains cultured in LB medium with 1% glycerol or 1% glucose. The *yfiQ* mutant exhibited changes in acetylation very similar to those in the wild-type strain in response to both carbon sources (Fig. 3C). In contrast, the *pta*-*ackA* double-knockout strain, which is defective in AcP synthesis, showed no significant increase in AceA acetylation upon supplementation with either glucose or glycerol (Fig. 3C). This phenomenon was consistent with the previous studies that the acetylation level was not increased in a *pta* mutant supplemented with glucose [12,26] Additionally, we compared the concentrations of AcP and acetate between these two mutants and the wild strain under the same culture conditions. The levels of AcP and acetate in the *yfiQ* mutant were similar to those of the wild strain when grown on the same carbon source. However, the *pta*-*ackA* double-knockout mutant showed obviously lower concentrations of both AcP and acetate compared to wild-type and *yfiQ* mutant strains (Fig. 3D). Moreover, carbon source-dependent changes in AceA acetylation correlated positively with the concentrations of AcP and acetate in both strains (Fig. 3C and D).

These results suggest that the acetyltransferase YfiQ is not responsible for carbon-dependent acetylation of AceA. Instead, it appears to rely primarily on glycolytic flux and subsequent accumulation of the metabolites, AcP and acetate, likely mediated by a non-enzymatic mechanism. The carbon source and its concentration are key factors influencing acetate metabolism in *E. coli*, which in turn affects intracellular levels of the acetyl donor AcP. Accordingly, the differential acetylation of AceA between glucose- and glycerol-grown cells is not attributable to carbon quantity, but rather to distinct metabolic outcomes: glucose metabolism generates significantly higher AcP levels than glycerol (Fig. 3D), and AcP serves as the primary mediator of non-enzymatic AceA acetylation. Thus, variations in downstream metabolite accumulation, rather than the carbon sources themselves, account for the observed acetylation differences.

In addition, acetate is reported as a major byproduct in microbial fermentation. Under aerobic conditions with glucose as the carbon source, *E. coli* exhibits rapid glucose uptake and metabolism, leading to significant acetate accumulation, which is known as overflow metabolism [31,32]. Lysine acetylation is known to vary dynamically with cellular growth phase, increasing markedly as cells transition into the stationary phase, a shift often correlated with acetate accumulation resulting from metabolic overflow [13]. In our study, the acetylation level of AceA exhibited a positive correlation with extracellular acetate concentration during the stationary phase. Thus, lysine acetylation serves as a regulatory response to the carbon flux and is intrinsically associated with overflow metabolism. In conclusion, our work mechanistically links these changes of carbon source to specific metabolites and demonstrates their functional consequences for the key metabolic enzyme of AceA. This correlation provides a predictive framework for choosing carbon source composition to achieve desired acetylation states, thereby offering a viable strategy to enhance the production of target compounds in metabolic engineering.

### 3.3 Acetylation decreases AceA activity

AceA can be acetylated by YfiQ-mediated enzymatic catalysis and AcP-mediated non-enzymatic mechanism. To evaluate the effect of acetylation on AceA function, we first measured its enzyme activity following in vitro treatments with YfiQ, CobB, and AcP, respectively. AceA activity was measured in buffer at pH 6.8, as described in previous studies [15]. To optimize the assay conditions, a range of enzyme concentrations (0.1–0.8 μM) was initially evaluated, and 0.4 μM AceA was selected as it provided a suitable balance between reaction rate and linearity (Supplementary Fig. 1A). It should be noted that, to ensure the reliability of AceA activity measurements, all activity assays were performed using freshly purified protein within 4–5 h of preparation, without freeze-thaw cycles or prolonged storage. A time-course stability assay also confirmed that AceA activity remained relatively stable within this timeframe, even though no protease inhibitors or DTT were added during purification (Supplementary Fig. 1B). AcP-induced acetylation inhibited AceA activity in a dose-dependent manner. After 120 min of treatment, the activity decreased to 62.6% with 10 mM AcP and 54.3% with 20 mM AcP (Fig. 1E). The enzyme activity of AceA was decreased by 45.4% with YfiQ and acetyl-CoA treatment, which enhanced AceA acetylation (Fig. 2E). In contrast, the treatment of CobB and NAD improved the AceA activity by 1.96-fold relative to the untreated control. The addition of NAM can inhibit CobB deacetylase activity and reduce the enzymatic activity of AceA, yet the remaining activity is still 1.36-fold higher than that of the untreated control (Fig. 2F). In addition, the addition of carbon sources such as glucose and glycerol enhances AceA acetylation by the non-enzymatic pathway. Thus, the AceA protein was purified in cells grown with varying concentrations of glucose or glycerol, and its activity was also measured. The observed increase in AceA acetylation upon glucose or glycerol supplementation correlated with a corresponding reduction in its enzymatic activity (Fig. 3E and F). Our findings revealed that variations in carbon source and concentration ultimately modulate AceA enzymatic activity by influencing its acetylation via a non-enzymatic mechanism, and thus established a clear mechanistic cascade linking carbon metabolism to post-translational modification and enzyme function. In summary, elevating the acetylation level of AceA regardless of the mechanism leads to the inhibition of its enzyme activity.

In addition, lysine acetylation has been reported to regulate the protein level by affecting the stability and half-life [[33], [34], [35], [36]]. Thus, the protein level and stability of AceA were assessed in wild-type, *ackA* knockout, and *pta*-*ackA* double mutant strains, which are characterized by distinct acetylation levels. The AceA protein levels showed no significant difference among the three strains after varying induction times. Similarly, when protein translation was inhibited by chloramphenicol, the rate of decrease in AceA levels over time was comparable across all strains (Supplementary Fig. 2). These results indicate that lysine acetylation has a minimal impact on both the expression and stability of the AceA protein.

Our findings demonstrated that AceA activity is reversibly controlled by lysine acetylation. This post-translational regulation offers unique advantages over transcriptional or translational control. It enables rapid, reversible, and precise regulation of enzyme activity in an energy-efficient manner, without the need for transcriptional reprogramming or protein synthesis/degradation.

AceA is the entry-point enzyme of the glyoxylate shunt, and regulating its acetylation status can influence the redirection of carbon flux between the glyoxylate shunt and TCA cycle. Thus, lysine acetylation can be used as a powerful tool in metabolic engineering to dynamically rewire central carbon metabolism and optimize the synthesis of target bioproducts derived from glyoxylate or acetyl-CoA by reversibly regulating the AceA activity.

### 3.4 Functional roles of specific lysine acetylation sites of AceA

Proteins often contain multiple lysine acetylation sites, each exerting distinct effects on enzyme activity depending on its spatial position. Consequently, the overall impact of lysine acetylation on enzyme activity arises from the combined contributions of all modified sites. Therefore, elucidating the specific acetylated lysine residues and characterizing their individual effects on enzyme activity could facilitate the precise and rational regulation of enzyme activity.

**Fig. 4:**
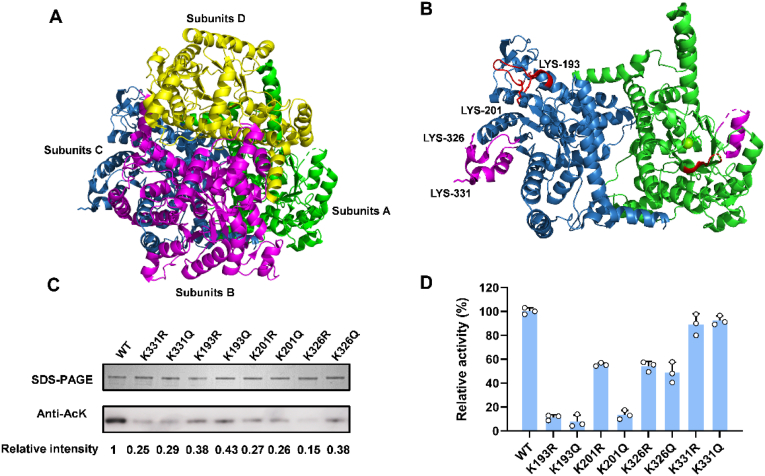
Functional roles of specific lysine acetylation sites on AceA enzyme activity. (A) Subunits A, B, C and D are shown in green, magenta, skyblue, and yellow cartoons, respectively. (B) Only subunits C (left) and A (right) of the whole tetramer are displayed. The segments 192-200 and 318-344 are highlighted in red and magenta, respectively, in both subunits. The unobserved residues in subunit A are replaced with dashed lines. K193, K201, K326, and K331 in subunit C are shown as sticks and labeled. (C) The acetylation levels of AceA mutants compared to wild-type. (D) The relative activity of AceA mutants compared to the wild-type.

11 acetylated lysine sites of AceA have been identified in the acetylome studies of *E. coli* using label-free mass spectrometry [12,13]. Based on the *E.* *coli* AceA structure, we first analyzed the spatial localization of the 11 acetylated lysine sites to make a preliminary assessment of which modifications were likely to affect enzyme activity. The structure of *E. coli* AceA was determined and analyzed by Britton et al. at 2.1 Å resolution (PDB ID: 1igw) [37]. This crystal belongs to the trigonal space group *P*3_2,_ housing a tetramer in the asymmetric unit (Fig. 4A). Subunits A and C of the tetramer are shown in Fig. 4B. Every subunit of the tetramer showed a similar *(β/α)*_*7*_*β* barrel with a disordered final helix to the C-terminus (residues 418–434), which is very close to the TIM barrel seen in many other enzymes. There are also two disordered segments (192–200 and 318–344) with several residues disappeared in subunits ABD but C, which indicates that these residues are highly flexible. Especially, these two segments lie at the C-terminal end of the barrel from the neighbor subunit, where glyoxylate is supposed to be bound [37,38]. The first disordered segment (192-200) is strongly conserved, which appears to act as a ‘lid’ and is subject to conformational changes. Among the 11 acetylated lysine sites, K193 in this region is conserved across all the 23 isocitrate lyases aligned, and K201 nearby this region is conserved across 22 of the 23 isocitrate lyases [37]. Another two identified acetylated lysine sites, K326 and K331, are in the other disordered segment (318–344), which is supposed to be important in the recognition of succinate with a conserved SPS motif (319–321) [37].

Based on our analysis, K193, K201, K326, and K331 are promising candidates whose acetylation may regulate AceA enzyme activity. We next focus on these four sites to study their individual effects on AceA activity and elucidate the underlying mechanism. Each of the four lysine residues was individually mutated to arginine (R) to maintain a positive charge and prevent acetylation, and glutamine (Q) to neutralize the charge and mimic the acetylated state, following the techniques commonly used in previous studies [[39], [40], [41]]. The purified wild-type AceA and all mutant proteins were assessed by SDS-PAGE. As shown in Supplementary Fig. 3A, all purified proteins exhibited high purity with a single predominant band corresponding to the expected molecular weight of AceA (∼47.5 kDa). By definition, the lysine-to-arginine/glutamine substitutions abolish the specific lysine residues targeted for acetylation, thereby resulting in an overall reduction of acetylation across all mutants (Fig. 4C). The enzyme activities of K193 mutants and K201Q decreased to below 20% of wild-type AceA, while the activities of K326 mutants and K201R were moderately reduced to about 50% activity (Fig. 4D). In contrast, the K331 mutation had no significant impact on activity, despite leading to a reduction in overall protein acetylation (Fig. 4D). The difference in these two segments (192–200, 318–344) between subunit C and subunits ABD of AceA indicates their high flexibility and may play important roles in enzyme activity. These results implied that the highly conserved segment (192–200) is vital to the enzyme activity. The acetylation of K193 and K201, which changed the electric charge, may affect the conformational changes of this highly flexible loop, affecting the opening or closure extent or efficiency of this ‘lid’. The mutations of K326 and K331 have a minor effect on the enzyme activity. Residues 319–324 are much more conserved than K326 and K331 [37], and may play the main role of the segment (318–344).

Based on our structural analysis and functional experiments, we have identified several key acetylated lysine sites that critically influence AceA enzyme activity. Through site-directed mutagenesis, we demonstrated that acetylation-mimetic mutations (K193Q, K201Q, and K326Q) significantly reduce the enzyme activity. This suggests that acetylation at these sites by neutralizing positive charge and increasing side chain bulk may thereby perturb the conformation and dynamics of these critical regions, and ultimately affect AceA activity. It is important to note that while arginine (R) substitutions preserve positive charge, the structural and chemical properties of the arginine side chain differ substantially from those of lysine. Unlike lysine's flexible and linear side chain, arginine possesses a larger, planar guanidinium group capable of forming distinctive hydrogen-bonding networks. Since these lysine residues are located in functionally critical and conformationally sensitive regions, thereby K193R, K201R, and K326R mutants also impair the enzyme activity of AceA. Our findings underscore the role of specific lysine acetylation in fine-tuning AceA activity by structurally and electrostatically modulating the flexible loop regions. In conclusion, our study enhances the understanding of post-translational regulation in the precision engineering of enzyme activity.

### 3.5 Rational regulation of AceA activity based on acetylation modification

Our above work identified several lysine residues on AceA whose acetylation negatively regulates enzymatic activity to varying degrees, and structural analysis provided initial mechanistic insights. AceA is the entry-point enzyme of the glyoxylate shunt, and enhancing its activity is a valuable strategy for improving acetate utilization, overcoming overflow metabolism, and increasing the carbon conversion efficiency in microbial synthesis. Based on these requirements of metabolic engineering, we also seek a rational design strategy centered on lysine acetylation to generate AceA variants with enhanced activity.

**Fig. 5:**
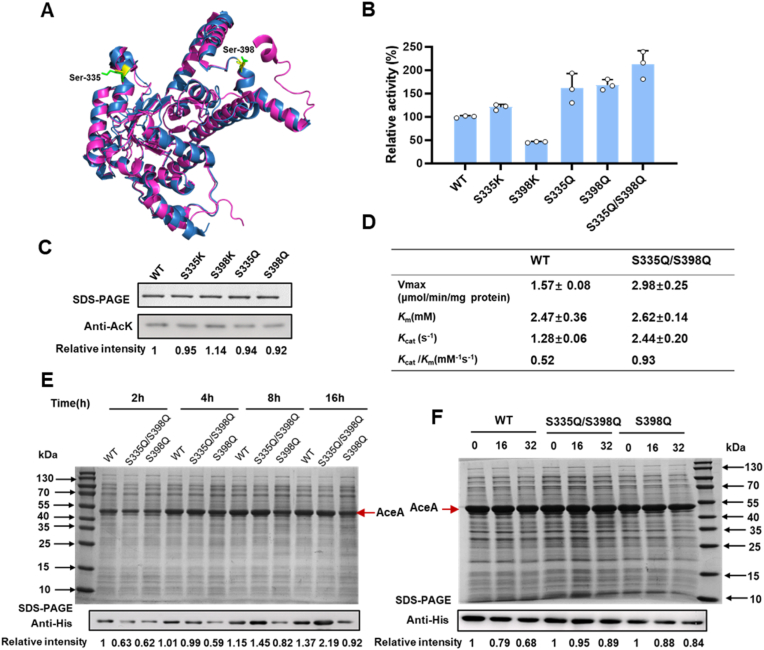
Acetylation-mediated rational regulation of AceA activity. (A) Structure alignment of EcAceA subunit C (skyblue) and MtAceA subunit C (magenta). S335 and S398 of EcAceA are shown in yellow sticks, and the corresponding K331 and K392 of MtAceA are shown in green sticks (PDB: 5DQL). [42] (B) The activity assay of S335 and S398 mutants. (C) The acetylation levels of S335 and S398 mutants. (D) Kinetic properties of AceA wild-type protein and S335Q/S398Q double mutant. Data represent mean ± standard deviation (n = 3). The protein expression (E) and stability (F) of the S398Q and S335Q/S398Q double mutant. AceA:47.5kD.

In *Mycobacterium tuberculosis* isocitrate lyase, lysine residues K331 and K392 were identified as acetylation sites. Substitution of these residues with glutamine, to mimic acetylation, was found to increase enzymatic activity approximately two-fold [15]. Alignment of *E. coli* AceA with *M. tuberculosis* isocitrate lyase revealed 62% amino acid sequence identity, and two key lysine residues, K331 and K392, in the *M. tuberculosis* correspond to serine residues, S335 and S398, in *E. coli* AceA (Supplementary Fig. 4). Moreover, structure alignment of isocitrate lyase from the two strains also showed high similarity (Fig. 5A). Given the significant sequence and structure conservation, we hypothesized that acetylation of K331 and K392 is functionally important in the *M. tuberculosis* isocitrate lyase, and that introducing lysines at the corresponding positions, S335 and S398, in *E. coli* AceA to promote the acetylation state might enhance enzymatic activity. To test this hypothesis, we introduced site-directed mutations, S335K and S398K, into *E. coli* AceA and assessed their impact on enzyme activity. Unexpectedly, the S335K mutation did not significantly enhance AceA activity, whereas the S398K mutant substantially reduced it compared to the wild-type (Fig. 5B). To directly mimic acetylation, we replaced both serines with glutamine. The resulting S335Q and S398Q mutants significantly improved the activity of AceA (Fig. 5B). We subsequently combined these beneficial mutations to generate the double mutants S335Q/S398Q. Notably, the S335Q/S398Q mutant achieved a 2.12-fold increase in the enzyme activity of *E.* *coli* AceA (Fig. 5B). It should be noted that all proteins used for these enzymatic activity assays exhibited high purity, as confirmed by SDS-PAGE analysis (Supplementary Fig. 3B).

To verify the substrate specificity of AceA and the S335Q/S398Q double mutant, control experiments were performed. When isocitrate was omitted or replaced with citrate, no significant AceA activity was detected in either the wild-type or the S335Q/S398Q double mutant. Similarly, replacing NADH with NADPH resulted in no detectable activity (Supplementary Fig. 5). These results confirm that both wild-type and the double mutant exhibit strict substrate specificity. In addition, the kinetic properties of AceA wild-type and S335Q/S398Q double mutant were also determined in *vitro*. Both wild-type and double mutant AceA followed Michaelis-Menten kinetics, and the kinetic parameters were determined using Lineweaver-Burk plots. The *K*_m_ values for isocitrate were similar between wild-type AceA and the double mutant, indicating that the double mutation does not significantly affect substrate affinity. However, the double mutant exhibited a 1.9-fold increase in *V*_max_, resulting in proportional enhancements in *K*_cat_ and overall catalytic efficiency (*K*_cat_/*K*_m_) compared to the wild-type enzyme (Fig. 5D). These results suggest that the enhanced catalytic efficiency of the double mutant is primarily attributable to an increased reaction rate rather than improved substrate binding.

As discussed above, segment (318-344) is disordered in three subunits of EcAceA, S335 mutations may influence the recognition of succinate [37]. The final helix to the C-terminus (residues 418–434) is also disordered but can be seen in the MtAceA structure. It can be found that K392 is near the final helix of MtAceA (Fig. 5A), where glyoxylate is supposed to be bound [42], indicating the corresponding S398 of EcAceA may influence the binding of glyoxylate. As acetylation of K331 and K392 can improve MtAceA activity [15], and the high sequential and structural similarities between EcAceA and MtAceA (Fig. 5A), it is reasonable that S335Q and S398Q can also improve the EcAceA activity. Unlike the glutamine mutants, the S335K and S398K mutations did not enhance AceA activity, likely because lysine residues (K335 and K398) cannot be acetylated in *E. coli.* Western blot analysis showed similar acetylation levels to those of wild-type AceA (Fig. 5C). Furthermore, the additional activity loss observed in the S398K mutant may result from the mutation interfering with substrate binding.

Beyond catalytic activity, the functional efficacy of a metabolic enzyme is critically influenced by its protein expression and stability [34,43]. We therefore systematically assessed these parameters for two high-activity mutants, S398Q and the double mutant S335Q/S398Q, in comparison to the wild-type AceA. Analysis of their expression profiles revealed that the S398Q single mutant consistently maintained lower abundance than the wild-type AceA. Strikingly, the S335Q/S398Q double mutant exhibited a progressive recovery of protein levels; after an initial decrease at 2 h, it rebounded to wild-type levels by 4 h and significantly exceeded them by 8 h (Fig. 5E). To assess stability, protein synthesis was inhibited by chloramphenicol after 4 h of induction with IPTG, and protein levels were detected at various time points. The S335Q/S398Q double mutant demonstrated markedly enhanced stability compared to the wild-type enzyme. While the wild-type AceA retained only 68% of its initial protein level, the double mutant maintained approximately 89% of its original quantity after 32 h of inhibition (Fig. 5F). This result indicates that the double mutation S335Q/S398Q significantly improves the protein's resistance to degradation. Collectively, our findings establish that the rationally designed S335Q/S398Q variant not only achieves superior catalytic efficiency but also exhibits improved molecular stability.

In conclusion, our study demonstrates the successful application of acetylation-mediated rational design to enhance both catalytic efficiency and stability of AceA. The engineered S335Q/S398Q double mutant represents a particularly remarkable outcome, exhibiting a more than 2-fold increase in enzymatic activity while simultaneously displaying improved protein stability. These combined traits make the S335Q/S398Q mutant a promising candidate for applications in metabolic engineering. In long-term fermentation processes, where protein degradation often limits pathway efficiency, the improved activity and stability of AceA can help maintain durable carbon flux through the glyoxylate shunt. This may be essential for enhancing acetate utilization and obtaining more efficient production.

### 3.6 Applications of AceA acetylation regulation in biosynthesis

Overflow metabolism, recognized as a major obstacle in *E. coli* bioprocessing, has attracted attention from biotechnologists and microbiologists for over four decades. Under fully aerated conditions, acetate accumulation accounts for 10∼30% of the carbon flux from glucose, thereby affecting the carbon atom economy. Furthermore, acetate accumulation also consumed a large amount of the acetyl-CoA pool, which may affect the production of acetyl-CoA-derived chemicals. Therefore. enhancing the carbon flux of the glyoxylate shunt may help improve the microbial synthesis of acetyl-CoA and glyoxylate-derived chemicals.

**Fig. 6:**
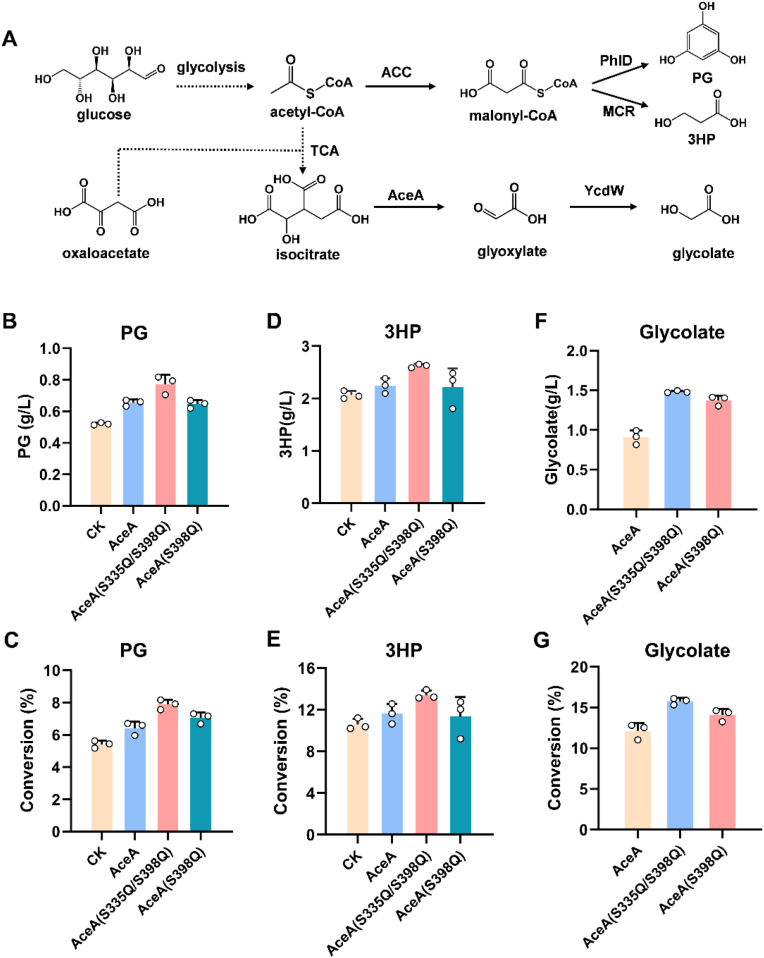
Applications of AceA mutants in the biosynthesis of PG, 3HP, and glycolate. (A) The biosynthesis pathway of PG, 3HP, and glycolate. The PG titer (B) and glucose conversion efficiency (C) in overexpressing the wild AceA and its mutant strains. The 3HP titer (D) and glucose conversion efficiency (E) in overexpressing the wild AceA and its mutant strains. The glycolate titer (F) and glucose conversion efficiency (G) in overexpressing the wild AceA and its mutant strains.

Phloroglucinol (PG) is a high-value chemical with applications as a pharmaceutical intermediate (e.g., muscle relaxants) and a key precursor for the production of agrochemicals and polymers. Its potent antioxidant properties also make it useful in cosmetics [44,45]. Three molecules of malonyl-CoA can be directly converted to PG by the catalysis of a type III polyketide synthase (PhlD) (Fig. 6A). By constructing an engineered strain co-expressing *phlD*, *marA*, and *ACCase*, Cao et al. achieved a phloroglucinol titer of 0.51 g/L in shake-flask cultivation [46]. Based on this strain, we further overexpressed AceA and its mutants, and the resulting strains were evaluated for PG production. Overexpression of AceA increased the PG titer to 0.65 g/L, indicating that reinforcing the glyoxylate shunt can enhance PG biosynthesis. A more substantial improvement was achieved by overexpressing the AceA(S335Q/S398Q) mutant, which elevated the PG concentration to 0.77 g/L. In contrast, the strain overexpressing the AceA(S398Q) single mutant produced a PG titer comparable to that of the wild-type AceA strain (Fig. 6B). The strain harboring the double mutant also achieved the highest glucose conversion efficiency among all the tested strains (Fig. 6C). 3-Hydroxypropionic acid (3HP) is recognized as a key platform chemical and ranks among the 12 most valuable biomass-derived chemicals listed by the U.S. Department of Energy [47]. The conversion of malonyl-CoA to 3HP via a two-step, NADPH-dependent reduction is catalyzed by malonyl-CoA reductase (MCR) from *Chloroflexus aurantiacus* [48]. Through directed evolution of the rate-limiting enzyme MCR-C and fine-tuning of MCR-N expression levels, Liu et al. achieved a significant improvement in enzyme activity [18]. The optimized MCR and *E. coli* ACCase were overexpressed to generate the production strain Q2191. Subsequently, AceA and its mutants were further overexpressed in the Q2191 background for 3HP production. Application of AceA acetylation regulation to 3HP biosynthesis showed an analogous effect to PG synthesis. AceA overexpression increased 3HP titer, and overexpressing the double mutant AceA(S335Q/S398Q) further increased both the 3HP titer and the conversion yield (Fig. 6D and E). These results are in full agreement with the functional characterization of the AceA mutants: the double mutant demonstrated superior activity, protein expression, and stability over the wild-type, whereas the S398Q mutant enhanced activity was offset by its significantly lower protein expression level.

Glycolate has significant commercial value in the cosmetics industry as a gentle exfoliator, in the industrial sector for cleaning and descaling, and most importantly, as a key monomer for producing biodegradable plastics like polyglycolic acid [49,50]. Glycolate is primarily bioproduced from the reduction of glyoxylate (Fig. 6A). Key metabolic engineering strategies include redirecting carbon flux from the TCA cycle to the glyoxylate shunt and introducing heterologous glyoxylate reductase [49,51]. AceA and its mutants were overexpressed in a strain with deletion of malate synthase (AceB) and overexpression of glyoxylate reductase (YcdW) to assess the glycolate production. The titers and glucose conversion efficiencies of glycolate in AceA mutant-overexpressing strains were higher than those of the AceA-overexpressing strain (Fig. 6F and G). In the double mutant strain, glycolate production reached 1.48 g/L, which was significantly higher than the 0.91 g/L observed in the AceA-overexpressing strain (Fig. 6F). In addition, all strains exhibited similar profiles in cell growth and glucose consumption during the process of PG, 3HP, and glycolate production, respectively (Supplementary Fig. 6).

Our findings demonstrated that engineering the glyoxylate shunt, particularly through the use of optimized AceA mutants, is a highly effective strategy for enhancing the production of both acetyl-CoA-derived and glyoxylate-derived chemicals. Overexpression of the AceA(S335Q/S398Q) double mutant to reinforce the glyoxylate shunt may increase the supply of key precursors for the synthesis of these target chemicals. Crucially, strengthening the glyoxylate shunt via AceA engineering also provides a metabolic bypass that diverts carbon away from the decarboxylative reactions of the TCA cycle. This reprogramming conserves carbon atoms that would otherwise be lost as CO_2_, thereby enhancing the carbon atom economy and directing more substrate toward product synthesis. Beyond these direct applications, our work suggests that such engineered AceA mutants could serve as a universal metabolic module. This module can be integrated into chassis strains designed for the microbial synthesis of a wide array of chemicals or bioplastics. Future efforts should explore integrating design informed by post-translational modification regulation with other protein engineering strategies, such as directed evolution, to generate AceA mutants with even higher activity.

## 4 Conclusions

The glyoxylate shunt serves as a crucial anaplerotic pathway, enabling efficient acetate assimilation and conservation of carbon skeletons by bypassing the decarboxylative steps of the TCA cycle. Isocitrate lyase (AceA) catalyzes the key step of this shunt, making its regulation pivotal for redirecting central carbon flux. In this study, we systematically characterized the lysine acetylation mechanism of *E. coli* AceA, and demonstrated its inhibitory effect on enzyme activity. Moreover, we developed an acetylation-mediated rational design strategy, creating a double-mutant AceA(S335Q/S398Q) with remarkably improved activity and stability. The effectiveness of this engineered enzyme was validated through the enhanced production of multiple chemicals, including PG, 3HP, and glycolate. Our study establishes the rational regulation of AceA acetylation as an effective and potential strategy to optimize the glyoxylate shunt, thereby enhancing carbon conversion efficiency and bioproduction in microbial synthesis. It will be a novel paradigm for using insights into the post-translational modification to rationally engineer metabolism.

## CRediT authorship contribution statement

**Ruoyu Jia:** Validation, Methodology, Investigation, Data curation, Conceptualization. **Jichao Wang:** Writing – original draft, Methodology, Funding acquisition, Data curation. **Ying Zhang:** Validation, Methodology, Data curation, Conceptualization. **Mo Xian:** Supervision, Investigation. **Qingsheng Qi:** Supervision, Investigation, Funding acquisition. **Min Liu:** Writing – review & editing, Writing – original draft, Methodology, Funding acquisition, Formal analysis, Data curation. **Guang Zhao:** Writing – review & editing, Supervision, Investigation, Funding acquisition, Data curation.

## Declaration of competing interest

The authors declare that they have no known competing financial interests or personal relationships that could have appeared to influence the work reported in this paper.

## References

[1] Liu M., Guo L., Fu Y., Huo M., Qi Q., Zhao G. (2021). Bacterial protein acetylation and its role in cellular physiology and metabolic regulation. Biotechnol Adv.

[2] Aggarwal S., Tolani P., Gupta S., Yadav A.K. (2021). Posttranslational modifications in systems biology. Adv Protein Chem Str.

[3] Minguez P., Parca L., Diella F., Mende D.R., Kumar R., Helmer-Citterich M., Gavin A.C., van Noort V., Bork P. (2012). Deciphering a global network of functionally associated post-translational modifications. Mol Syst Biol.

[4] Ren J., Sang Y., Lu J., Yao Y.F. (2017). Protein acetylation and its role in bacterial virulence. Trends Microbiol.

[5] Verdin E., Ott M. (2013). Acetylphosphate: a novel link between lysine acetylation and intermediary metabolism in bacteria. Mol Cell.

[6] AbouElfetouh A., Kuhn M.L., Hu L.I., Scholle M.D., Sorensen D.J., Sahu A.K., Becher D., Antelmann H., Mrksich M., Anderson W.F. (2015). The *E. coli* sirtuin CobB shows no preference for enzymatic and nonenzymatic lysine acetylation substrate sites. Microbiologyopen.

[7] Phillips D.M. (1963). The presence of acetyl groups of histones. Biochem J.

[8] Verdin E., Ott M. (2015). 50 years of protein acetylation: from gene regulation to epigenetics, metabolism and beyond. Nat Rev Mol Cell Biol.

[9] Shvedunova M., Akhtar A. (2022). Modulation of cellular processes by histone and non-histone protein acetylation. Nat Rev Mol Cell Biol.

[10] Mdluli K., Spigelman M. (2006). Novel targets for tuberculosis drug discovery. Curr Opin Pharmacol.

[11] Sarkhel R., Apoorva S., Priyadarsini S., Sridhar H.B., Bhure S.K., Mahawar M. (2022). Malate synthase contributes to the survival of *Salmonella* Typhimurium against nutrient and oxidative stress conditions. Sci Rep.

[12] Kuhn M.L., Zemaitaitis B., Hu L.I., Sahu A., Sorensen D., Minasov G., Lima B.P., Scholle M., Mrksich M., Anderson W.F. (2014). Structural, kinetic and proteomic characterization of acetyl phosphate-dependent bacterial protein acetylation. PLoS One.

[13] Schilling B., Christensen D., Davis R., Sahu A.K., Hu L.I., Walker-Peddakotla A., Sorensen D.J., Zemaitaitis B., Gibson B.W., Wolfe A.J. (2015). Protein acetylation dynamics in response to carbon overflow in *Escherichia coli*. Mol Microbiol.

[14] Baba T., Ara T., Hasegawa M., Takai Y., Okumura Y., Baba M., Datsenko K.A., Tomita M., Wanner B.L., Mori H. (2006). Construction of *Escherichia coli* K-12 in-frame, single-gene knockout mutants: the Keio collection. Mol Syst Biol.

[15] Bi J., Wang Y., Yu H., Qian X., Wang H., Liu J., Zhang X. (2017). Modulation of central carbon metabolism by acetylation of isocitrate lyase in *Mycobacterium tuberculosis*. Sci Rep.

[16] Wang Q., Zhang Y., Yang C., Xiong H., Lin Y., Yao J., Li H., Xie L., Zhao W., Yao Y. (2010). Acetylation of metabolic enzymes coordinates carbon source utilization and metabolic flux. Science.

[17] Cao Y.J., Jiang X.L., Zhang R.B., Xian M. (2011). Improved phloroglucinol production by metabolically engineered *Escherichia coli*. Appl Microbiol Biotechnol.

[18] Liu C., Ding Y., Zhang R., Liu H., Xian M., Zhao G. (2016). Functional balance between enzymes in malonyl-CoA pathway for 3-hydroxypropionate biosynthesis. Metab Eng.

[19] Liu M., Ding Y., Xian M., Zhao G. (2018). Metabolic engineering of a xylose pathway for biotechnological production of glycolate in *Escherichia coli*. Microb Cell Fact.

[20] Wang Q., Xu J., Sun Z., Luan Y., Li Y., Wang J., Liang Q., Qi Q. (2019). Engineering an in vivo EP-bifido pathway in *Escherichia coli* for high-yield acetyl-CoA generation with low CO_2_ emission. Metab Eng.

[21] Wolfe A.J. (2016). Bacterial protein acetylation: new discoveries unanswered questions. Curr Genet.

[22] VanDrisse C.M., Escalante-Semerena J.C. (2019). Protein acetylation in bacteria. Annu Rev Microbiol.

[23] Klein A.H., Shulla A., Reimann S.A., Keating D.H., Wolfe A.J. (2007). The intracellular concentration of acetyl phosphate in *Escherichia coli* is sufficient for direct phosphorylation of two-component response regulators. J Bacteriol.

[24] Ren J., Sang Y., Qin R., Su Y., Cui Z., Mang Z., Li H., Lu S., Zhang J., Cheng S. (2019). Metabolic intermediate acetyl phosphate modulates bacterial virulence via acetylation. Emerg Microb Infect.

[25] Hentchel K.L., Escalante-Semerena J.C. (2015). Acylation of biomolecules in prokaryotes: a widespread strategy for the control of biological function and metabolic stress. Microbiol Mol Biol Rev.

[26] Weinert B.T., Iesmantavicius V., Wagner S.A., Schölz C., Gummesson B., Beli P., Nyström T., Choudhary C. (2013). Acetyl-phosphate is a critical determinant of lysine acetylation in *E. coli*. Mol Cell.

[27] Xu Z., Zhang H., Zhang X., Jiang H., Liu C., Wu F., Qian L., Hao B., Czajkowsky D.M., Guo S. (2019). Interplay between the bacterial protein deacetylase CobB and the second messenger c-di-GMP. EMBO J.

[28] Tu S., Guo S.J., Chen C.S., Liu C.X., Jiang H.W., Ge F., Deng J.Y., Zhou Y.M., Czajkowsky D.M., Li Y. (2015). YcgC represents a new protein deacetylase family in prokaryotes. eLife.

[29] Tan Y.C., Xu Z.H., Tao J., Ni J.J., Zhao W., Lu J., Yao Y.F. (2016). A SIRT4-like auto ADP-ribosyltransferase is essential for the environmental growth of *Mycobacterium smegmatis*. Acta Biochim Biophys Sin.

[30] Schilling B., Basisty N., Christensen D.G., Sorensen D., Orr J.S., Wolfe A.J., Rao C.V. (2019). Global lysine acetylation in *Escherichia coli* results from growth conditions that favor acetate fermentation. J Bacteriol.

[31] Liu M., Feng X., Ding Y., Zhao G., Liu H., Xian M. (2015). Metabolic engineering of *Escherichia coli* to improve recombinant protein production. Appl Microbiol Biotechnol.

[32] Bernal V., Castano-Cerezo S., Canovas M. (2016). Acetate metabolism regulation in *Escherichia coli*: carbon overflow, pathogenicity, and beyond. Appl Microbiol Biotechnol.

[33] Kuczynska-Wisnik D., Moruno-Algara M., Stojowska-Swedrzynska K., Laskowska E. (2016). The effect of protein acetylation on the formation and processing of inclusion bodies and endogenous protein aggregates in *Escherichia coli* cells. Microb Cell Fact.

[34] Sang Y., Ren J., Qin R., Liu S.T., Cui Z.L., Cheng S., Liu X.Y., Lu J., Tao J., Yao Y.F. (2017). Acetylation regulating protein stability and DNA-binding ability of HilD, thus modulating *Salmonella* Typhimurium virulence. J Infect Dis.

[35] Sang Y., Ren J., Ni J., Tao J., Lu J., Yao Y.F. (2016). Protein acetylation is involved in *Salmonella enterica* serovar typhimurium virulence. J Infect Dis.

[36] Liu M., Huo M., Guo L., Fu Y., Xian M., Qi Q., Liu W., Zhao G. (2022). Lysine acetylation decreases enzyme activity and protein level of *Escherichia coli* lactate dehydrogenase. Eng Microb.

[37] Britton K., Abeysinghe I., Baker P., Barynin V., Diehl P., Langridge S., McFadden B., Sedelnikova S., Stillman T., Weeradechapon K. (2001). The structure and domain organization of *Escherichia coli* isocitrate lyase. Acta Crystallogr D Biol Crystallogr.

[38] Britton K., Langridge S., Baker P., Weeradechapon K., Sedelnikova S., De Lucas J., Rice D., Turner G. (2000). The crystal structure and active site location of isocitrate lyase from the fungus *Aspergillus nidulans*. Structure.

[39] Zhang Q., Zhou A., Li S., Ni J., Tao J., Lu J., Wan B., Li S., Zhang J., Zhao S. (2016). Reversible lysine acetylation is involved in DNA replication initiation by regulating activities of initiator DnaA in *Escherichia coli*. Sci Rep.

[40] Sun M., Guo H., Lu G., Gu J., Wang X., Zhang X.E., Deng J. (2016). Lysine acetylation regulates the activity of *Escherichia coli* S-adenosylmethionine synthase. Acta Biochim Biophys Sin.

[41] Zhao D., Zou S.W., Liu Y., Zhou X., Mo Y., Wang P., Xu Y.H., Dong B., Xiong Y., Lei Q.Y. (2013). Lysine-5 acetylation negatively regulates lactate dehydrogenase A and is decreased in pancreatic cancer. Cancer Cell.

[42] Pham T.V., Murkin A.S., Moynihan M.M., Harris L., Tyler P.C., Shetty N., Sacchettini J.C., Huang H-l, Meek T.D. (2017). Mechanism-based inactivator of isocitrate lyases 1 and 2 from *Mycobacterium tuberculosis*. Proc Natl Acad Sci U S A.

[43] Liu L., Yang H., Shin H-d, Chen R.R., Li J., Du G., Chen J. (2013). How to achieve high-level expression of microbial enzymes: strategies and perspectives. Bioengineered.

[44] Rao G., Lee J.K., Zhao H. (2013). Directed evolution of phloroglucinol synthase PhlD with increased stability for phloroglucinol production. Appl Microbiol Biotechnol.

[45] Mitchell A.R., Coburn M.D., Schmidt R.D., Pagoria P.F., Lee G.S. (2002). Advances in the chemical conversion of surplus energetic materials to higher value products. Thermochim Acta.

[46] Cao Y., Jiang X., Zhang R., Xian M. (2011). Improved phloroglucinol production by metabolically engineered *Escherichia coli*. Appl Microbiol Biotechnol.

[47] Werpy T., Petersen G. (2004). Top value added chemicals from biomass.

[48] Hügler M., Menendez C., Schägger H., Fuchs G. (2002). Malonyl-coenzyme A reductase from *Chloroflexus aurantiacus*, a key enzyme of the 3-hydroxypropionate cycle for autotrophic CO_2_ fixation. J Bacteriol.

[49] Yu Y., Shao M., Li D., Fan F., Xu H., Lu F., Bi C., Zhu X., Zhang X. (2020). Construction of a carbon-conserving pathway for glycolate production by synergetic utilization of acetate and glucose in *Escherichia coli*. Metab Eng.

[50] Zhan T., Chen Q., Zhang C., Bi C., Zhang X. (2020). Constructing a novel biosynthetic pathway for the production of glycolate from glycerol in *Escherichia coli*. ACS Synth Biol.

[51] Deng Y., Ma N., Zhu K., Mao Y., Wei X., Zhao Y. (2018). Balancing the carbon flux distributions between the TCA cycle and glyoxylate shunt to produce glycolate at high yield and titer in *Escherichia coli*. Metab Eng.

