# Supplementary material for: The acetylation mechanism and engineering strategy of isocitrate lyase for optimizing biosynthesis in Escherichia coli
Source: Synth Syst Biotechnol. 2026 May 23;14:310–20. doi: 10.1016/j.synbio.2026.04.031 (PMC13224001; doi:10.1016/j.synbio.2026.04.031)
Supplement: Multimedia component 1 [file mmc1.docx]

**Supplementary Table 1**  Strains and plasmids used in this study

| **Plasmids and Strains** | **Description** | | **Source** |
| --- | --- | --- | --- |
| **Plasmids** |  |  | |
| pACYCDuet1 | Cm^r^ oriP_15A_ lacI^q^ P_T7_ | Novagen | |
| pETDuet1 | Amp_r_ oriP_BR322_ lacIq P_T7_ | Novagen | |
| pACYCDuet1- *patZ* | Cm^r^ oriP_15A_ lacI^q^ P_T7_ *patZ* | [35] | |
| pACYCDuet1- *cobB* | Cm^r^ oriP_15A_ lacI^q^ P_T7_ *cobB* | [35] | |
| pETDuet1- *aceA* | Ampr oriPBR322 lacIq PT7 *aceA* | This study | |
| pETDuet-*aceA*(K193R) | Ampr oriPBR322 lacIq PT7 *aceA*(K193R) | This study | |
| pETDuet-*aceA*(K193Q) | Ampr oriPBR322 lacIq PT7 *aceA*(K193Q) | This study | |
| pETDuet-*aceA*(K201R) | Ampr oriPBR322 lacIq PT7 *aceA*(K201Q) | This study | |
| pETDuet-*aceA*(K201Q) | Ampr oriPBR322 lacIq PT7 *aceA*(K201Q) | This study | |
| pETDuet-*aceA*(K326R) | Ampr oriPBR322 lacIq PT7 *aceA*(K326R) | This study | |
| pETDuet-*aceA*(K326Q) | Ampr oriPBR322 lacIq PT7 *aceA*(K326Q) | This study | |
| pETDuet-*aceA*(K331R) | Ampr oriPBR322 lacIq PT7 *aceA*(K331R) | This study | |
| pETDuet-*aceA*(K331Q) | Ampr oriPBR322 lacIq PT7 *aceA*(K331Q) | This study | |
| pETDuet-*aceA*(S335K) | Ampr oriPBR322 lacIq PT7 *aceA*(S335K) | This study | |
| pETDuet-*aceA*(S335Q) | Ampr oriPBR322 lacIq PT7 *aceA*(S335Q) | This study | |
| pETDuet-*aceA*(S398K) | Ampr oriPBR322 lacIq PT7 *aceA*(S398K) | This study | |
| pETDuet-*aceA*(S398Q) | Amp_r_ oriP_BR322_ lacIq P_T7_ *aceA*(S398Q) | This study | |
| pETDuet-*aceA*(S335Q/S398Q) | Amp_r_ oriP_BR322_ lacIq P_T7_ *aceA*(S335Q/S398Q) | This study | |
| pET28a-*phlD*-*marA* | Kan^r^ oriP_BR322_ lacIq P_T7_ *phlD* _PT7_ *marA* | [16] | |
| pACYCDuet1-*acc* | rep_p15A_ Cm^R^ lacI P_T7_ *accA* _PT7_ *accD* _PT7_ *accBC* | [16] | |
| pETDuet1-*mcr* | rep_pBR322_ Amp^R^ *lacI* P_lac,P2-51_*mcr*_1-549_ P_T7_ *mcr*_550-1219_ (N940V/K1106W/S1114R) | [17] | |
| pET28a-*phlD*-*marA*-*aceA* | Kan^r^ oriP_BR322_ lacIq P_T7_ *phlD* _PT7_ *marA aceA* | This study | |
| pET28a-*phlD*-*marA*-*aceA*(S398Q) | Kan^r^ oriP_BR322_ lacIq P_T7_ *phlD* _PT7_ *marA aceA*(K398Q) | This study | |
| pET28a-*phlD*-*marA*-*aceA*(S335Q/S398Q) | Kan^r^ oriP_BR322_ lacIq P_T7_ *phlD* _PT7_ *marA aceA*(S335Q/S398Q) | This study | |
| pACYCDuet1-*acc-aceA* | rep_p15A_ Cm^R^ lacI P_T7_ *accA* _PT7_ *accD* _PT7_ *accBC* | This study | |
| pACYCDuet1-*acc-aceA*(S398Q) | rep_p15A_ Cm^R^ lacI P_T7_ *accA* _PT7_ *accD* _PT7_ *accBC-aceA*(S398Q) | This study | |
| pACYCDuet1-*acc-aceA*(S335Q/S398Q) | rep_p15A_ Cm^R^ lacI P_T7_ *accA* _PT7_ *accD* _PT7_ *accBC- aceA*(S335Q/S398Q) | This study | |
|  |  | This study | |
| pACYCDuet1-*ycdw* | Cm^r^ oriP_15A_ lacI_q_ P_T7_ *ycdw* | This study | |
| **Strains** |  |  | |
| *E. coli* DH5α | F^–^ *supE44ΔlacU169*(𝜙80 *lacZ ΔM15*)*hsdR17 recA1 endA1 gyrA96 thi-1 relA1* | Invitrogen | |
| **Strains used for the experiments on the acetylation mechanisms of AceA** | | | |
| *E. coli* BL21(DE3) | F^–^ *ompT* *gal dcm lon hsdSB* (rB^–^ mB^–^) 𝜆(DE3) | Invitrogen | |
| Q3509 | *E. coli* BL21(DE3)/ pACYCDuet1- *patZ* | [35] | |
| Q3510 | *E. coli* BL21(DE3)/ pACYCDuet1- *cobB* | [35] | |
| Q5795 | *E. coli* BL21(DE3) *ΔcobB*/ pETDuet1- *aceA* | This study | |
| Q5796 | *E. coli* BL21(DE3) *ΔpatZ*/ pETDuet1- *aceA* | This study | |
| Q5797 | *E. coli* BL21(DE3) *Δpta*/ pETDuet1- *aceA* | This study | |
| Q5798 | *E. coli* BL21(DE3) *ΔackA/* pETDuet1- *aceA* | This study | |
| Q5799 | *E. coli* BL21(DE3) *ΔackAΔpta*/ pETDuet1- *aceA* | This study | |
| **Strains used for the experiments on the AceA mutants** | | | |
| Q7086 | *E. coli* BL21(DE3)/pETDuet-*aceA*(K331R) | This study | |
| Q7087 | *E. coli* BL21(DE3)/pETDuet-*aceA*(K331Q) | This study | |
| Q7088 | *E. coli* BL21(DE3)/pETDuet-*aceA*(K193R) | This study | |
| Q7089 | *E. coli* BL21(DE3)/pETDuet-*aceA*(K193Q) | This study | |
| Q7090 | *E. coli* BL21(DE3)/pETDuet-*aceA*(K201R) | This study | |
| Q7091 | *E. coli* BL21(DE3)/pETDuet-*aceA*(K201Q) | This study | |
| Q7092 | *E. coli* BL21(DE3)/pETDuet-*aceA*(K326R) | This study | |
| Q7093 | *E. coli* BL21(DE3)/pETDuet-*aceA*(K326Q) | This study | |
| Q7094 | *E. coli* BL21(DE3)/pETDuet-*aceA*(S335K) | This study | |
| Q7096 | *E. coli* BL21(DE3)/pETDuet-*aceA*(S335Q) | This study | |
| Q7097 | *E. coli* BL21(DE3)/pETDuet-*aceA*(S398K) | This study | |
| Q7099 | *E. coli* BL21(DE3)/pETDuet-*aceA*(S398Q) | This study | |
| Q7100 | *E. coli* BL21(DE3)/pETDuet-*aceA*(S398Q/S335Q) | This study | |
| **The glycolate-producing strains** | | | |
| Q7103 | *E. coli* BL21(DE3) *ΔaceB* | This study | |
| Q7408 | *E. coli* BL21(DE3) *ΔaceB* /pETDuet-*aceA*/ pACYCDuet1-*ycdw* | This study | |
| Q7104 | *E. coli* BL21(DE3) *ΔaceB* /pETDuet-*aceA*(S398Q/S335Q)/ pACYCDuet1-*ycdw* | This study | |
| Q7105 | *E. coli* BL21(DE3) *ΔaceB*/pETDuet-*aceA*(S398Q)/ pACYCDuet1-*ycdw* | This study | |
| **The PG-producing strains** | | | |
| Q1944 | *E. coli* BL21(DE3)/ pET28a-*phlD*-*marA* / pACYCDuet1-*acc* | [16] | |
| Q7108 | *E. coli* BL21(DE3)/ pET28a-*phlD*-*marA*-*aceA*/ pACYCDuet1-*acc* | This study | |
| Q7107 | *E. coli* BL21(DE3)/ pET28a-*phlD*-*marA*-*aceA*(S398Q/S335Q)/ pACYCDuet1-*acc* | This study | |
| Q7106 | *E. coli* BL21(DE3)/ pETDuet-*phlD-marA-aceA*(S398Q)/ pACYCDuet1-*acc* | This study | |
| **The 3HP-producing strains** | | | |
| Q2191 | *E. coli* BL21(DE3)/ pETDuet-*mcr* / pACYCDuet1-*acc* | This study | |
| Q7405 | *E. coli* BL21(DE3)/ pETDuet-*mcr* / pACYCDuet1-*acc-aceA* | This study | |
| Q7406 | *E. coli* BL21(DE3)/ pETDuet-*mcr* / pACYCDuet1-*acc-aceA* (S335Q/S398Q) | This study | |
| Q7407 | *E. coli* BL21(DE3)/ pETDuet-*mcr* / pACYCDuet1-*acc-aceA* (S398Q) | This study | |

**
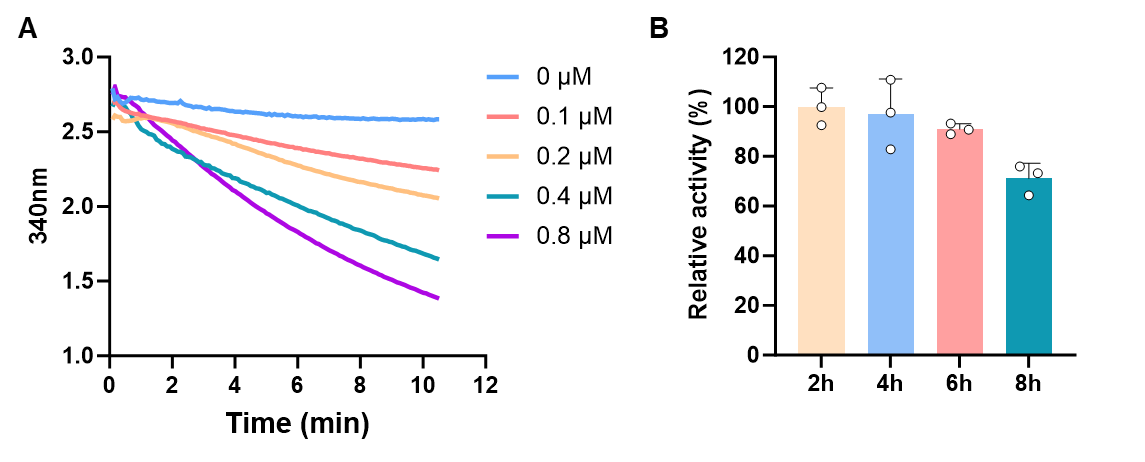
**

**Supplementary Fig. 1. Optimization and stability assessment of AceA for activity assays.** (A) Optimization of AceA concentration for activity assays. (B) Time-course of AceA activity after isolation for different time. The purified protein was stored in PBS buffer (pH 7.4) without protease inhibitors or DTT. Data represent mean ± SD from three independent experiments.


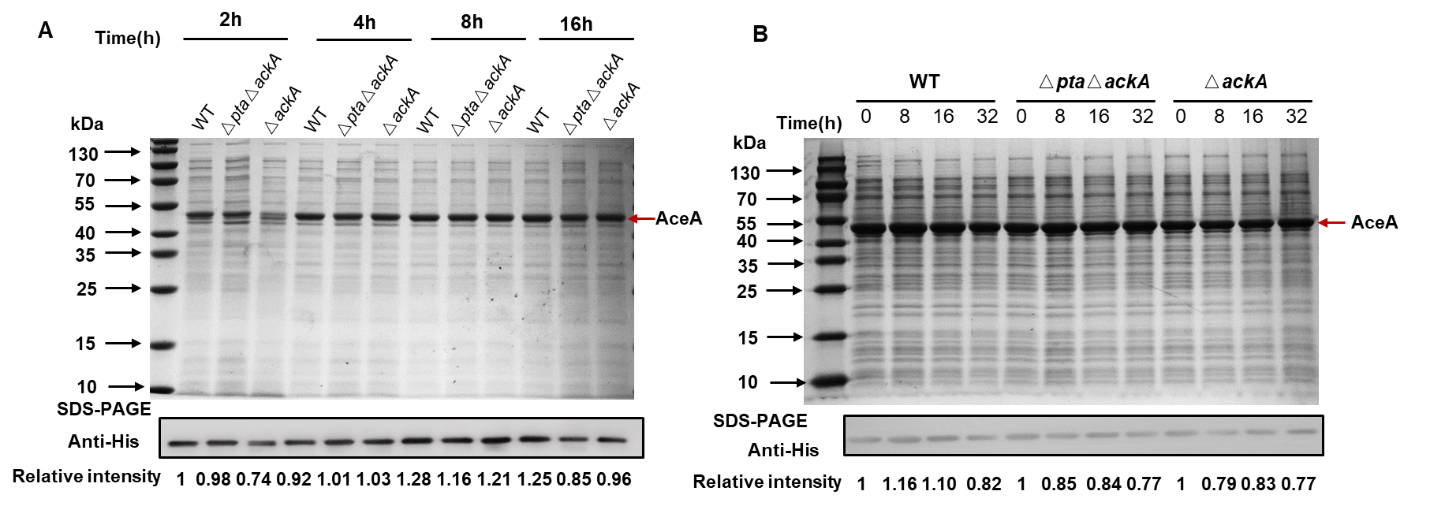


**Supplementary Fig. 2. The effects of lysine acetylation on the *E. coli* AceA protein expression and stability.** (A) The protein expression of AceA in the *ackA* and *pta-ackA* double mutant after varying induction times. (B) The protein stability of AceA in the *ackA* and *pta-ackA* double mutant. AceA：47.5kD


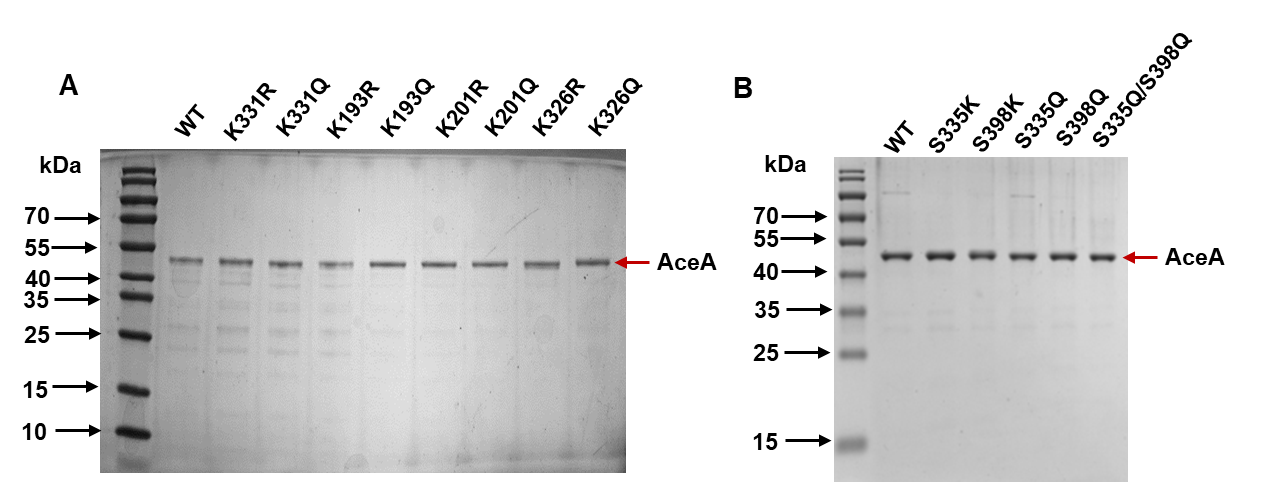


**Supplementary Fig. 3. SDS-PAGE analysis of purified wild-type AceA and mutant proteins.** (A) Purity assessment of wild-type AceA and acetylation-mimetic mutants. (B) Purity assessment of wild-type AceA and rationally designed mutants.


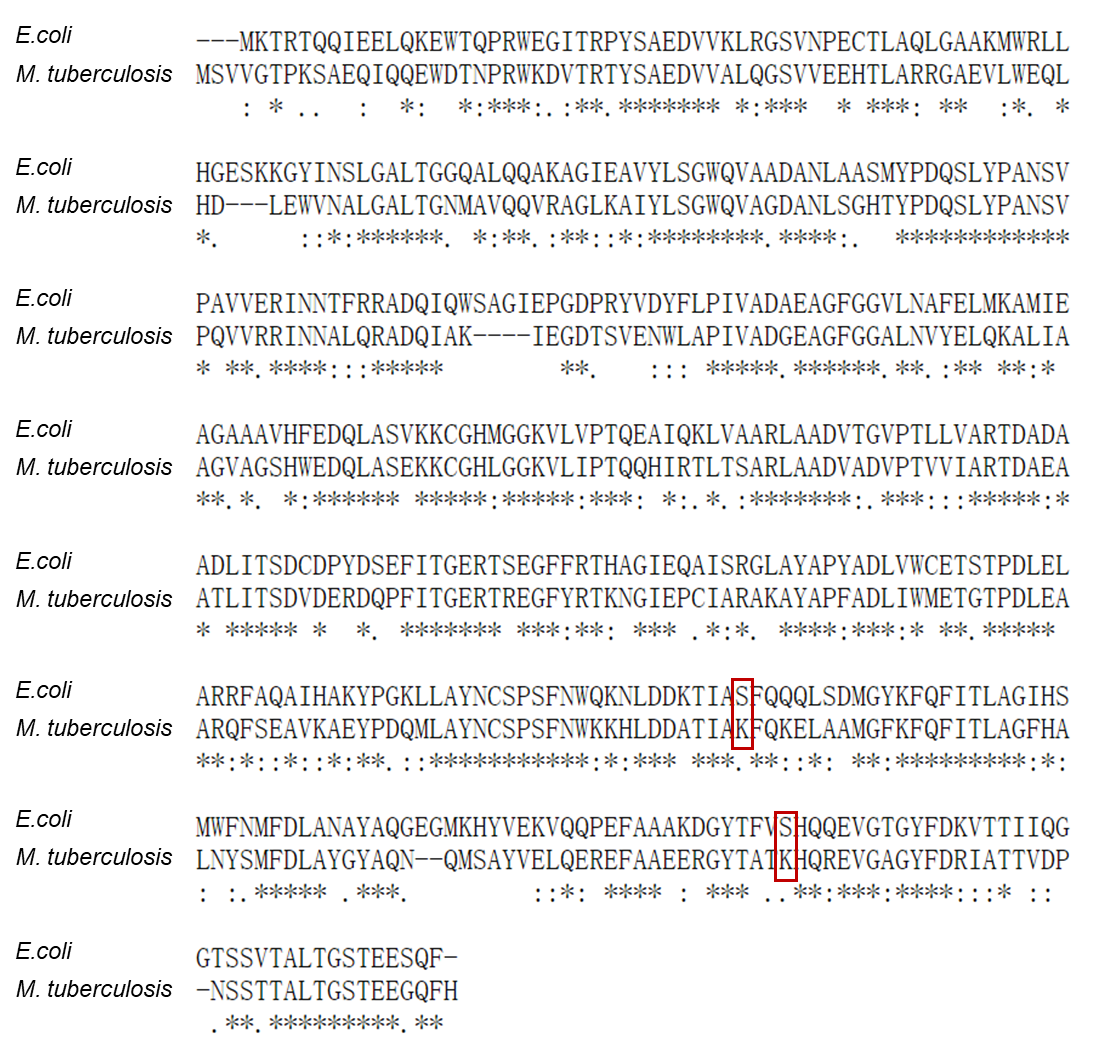


**Supplementary Fig. 4. Alignment of *E. coli* AceA with *M. tuberculosis* isocitrate lyase**


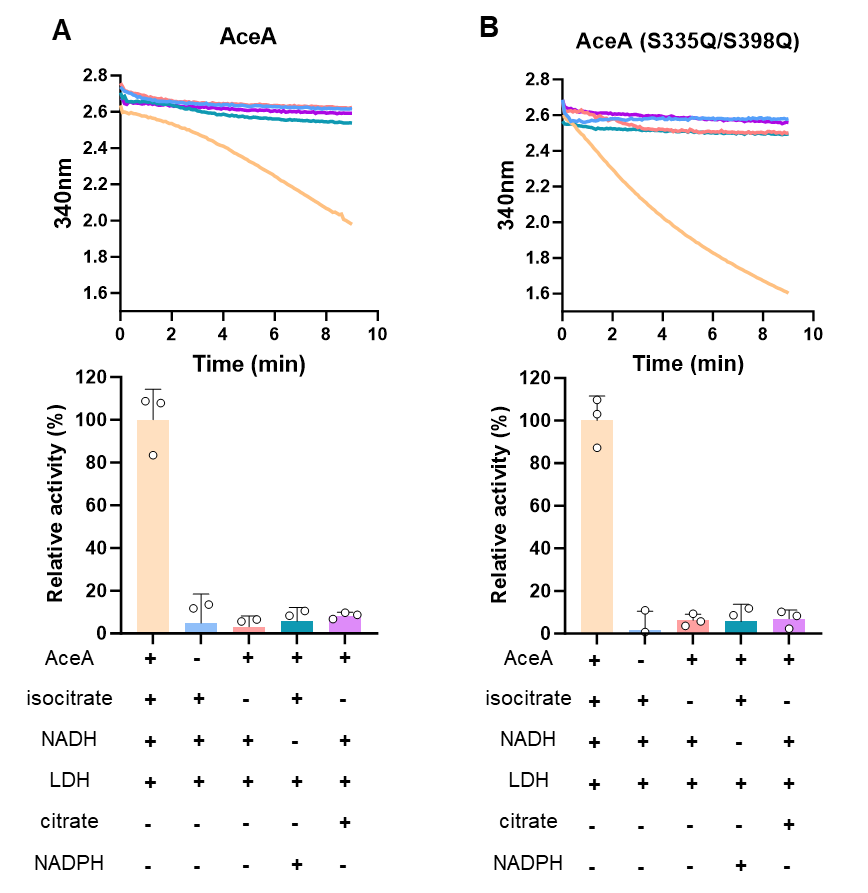


**Supplementary Fig. 5. Substrate specificity of wild-type AceA and** **the S335Q/S398Q double mutant.** (A) Time-course of absorbance changes at 340 nm and relative activity for wild-type AceA under different assay conditions. (B) Time-course of absorbance changes at 340 nm and relative activity for the S335Q/S398Q double mutant under different assay conditions.


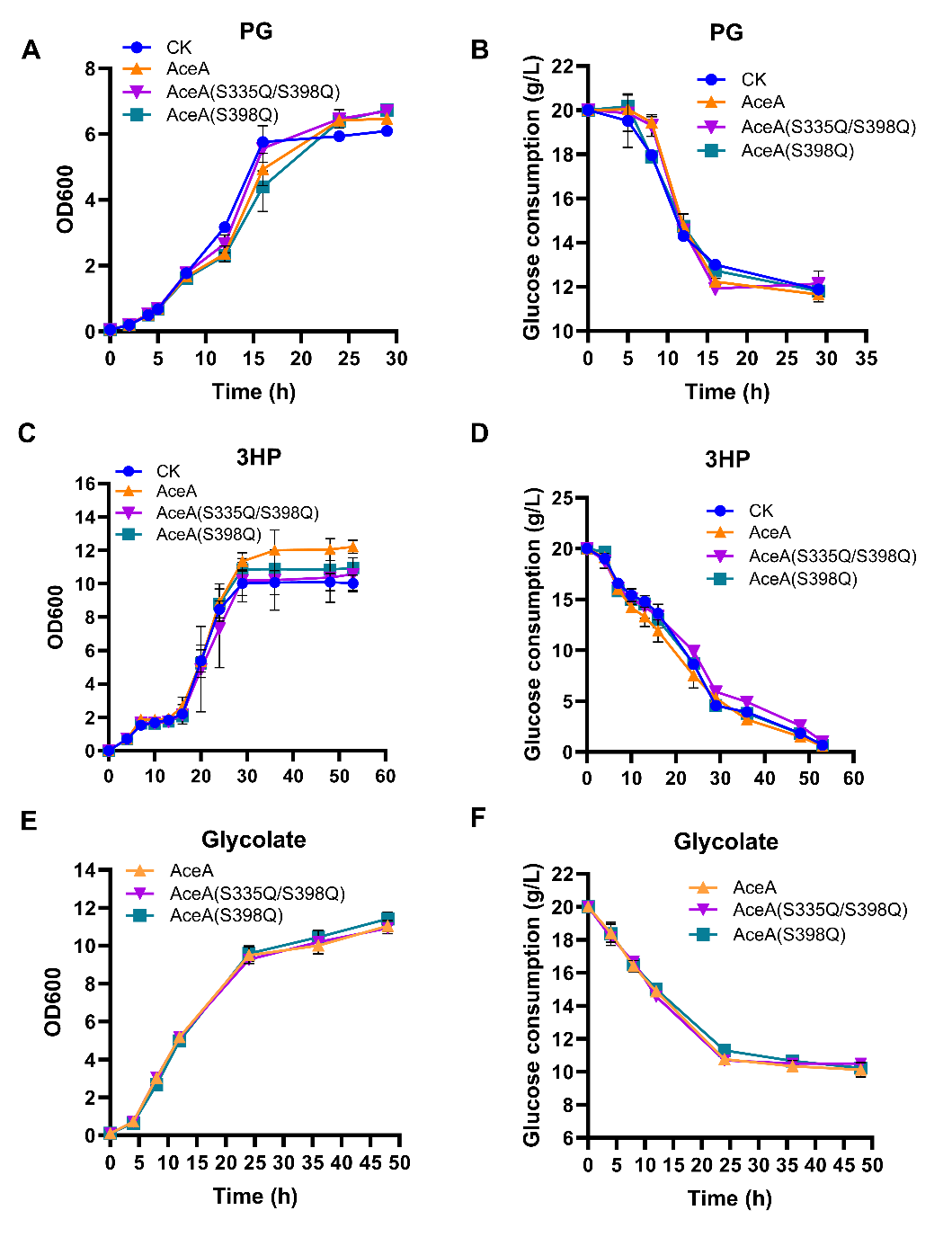


**Supplementary Fig. 6. Applications of AceA mutants in the biosynthesis of PG, 3HP and glycolate.** Cell growth (A) and glucose consumption (B) of the wild AceA and its overexpressed mutant strains in PG production. Cell growth (C) and glucose consumption (D) of the wild AceA and its overexpressed mutant strains in 3HP production. Cell growth (E) and glucose consumption (F) of the wild AceA and its overexpressed mutant strains in glycolate production.
